# THE ASSOCIATION BETWEEN APOE Ε4 AND EPISODIC MEMORY IN MIDLIFE: TESTING THE COGNITIVE PHENOTYPE HYPOTHESIS

**DOI:** 10.1093/geroni/igad104.3626

**Published:** 2023-12-21

**Authors:** Kyoung Shin Park, Jennifer Etnier, Samantha DuBois, Samuel Kibildis, Hadassah Som-Pimpong, Jarod Vance, Christopher Wahlheim

**Affiliations:** University of North Carolina at Greensboro, Greensboro, North Carolina, United States; University of North Carolina at Greensboro, Greensboro, North Carolina, United States; University of North Carolina at Greensboro, Greensboro, North Carolina, United States; University of North Carolina at Greensboro, Greensboro, North Carolina, United States; University of North Carolina at Greensboro, Greensboro, North Carolina, United States; North Carolina Institute for Medical Research, Inc, Durham, North Carolina, United States; University of North Carolina at Greensboro, Greensboro, North Carolina, United States

## Abstract

Carrying at least one ε4 allele of Apolipoprotein E (APOE ε4) is a strong genetic risk factor for Alzheimer’s disease (AD). It is contentious whether the effect of APOE ε4 on memory decline is because of cognitive phenotype or prodromal AD cases. Inconsistent findings on this topic could be due to the presence of AD pathology in older ages and the lack of sensitivity of memory measures. To exclude potential prodromal AD cases, we tested the association between APOE ε4 and episodic memory among cognitively unimpaired adults in midlife. Data from a subset of 103 participants (Mage=57±6 years; 87.4% females) from an ongoing clinical trial (NIH R01AG058919) were analyzed. APOE genotype was assessed from passive drool saliva samples, and participants completed memory assessments. Analyses of covariance compared 39 carriers of APOE ε4 versus 64 non-carriers of APOE ε4 for their performance of mnemonic discrimination, auditory-verbal and visuospatial short- and long-term memory, paired associative memory, and verbal logical memory, while controlling relevant covariates (e.g., age, education, sex, and physical activity level). After covariate adjustments, significant negative relationships between APOE ε4 and mnemonic discrimination were observed while nearly significant negative relationships were observed for auditory-verbal long-term memory. These findings imply that APOE ε4 could be associated with a cognitive phenotype of decline in mnemonic discrimination and auditory-verbal long-term memory, and the phenotype effects on memory decline could start in midlife. This suggests the importance of early lifestyle interventions for cognitive health in midlife, especially in people at genetic risk for AD.

